# Global Epidemiology of Smoking and Liver Cancer from 1990 to 2021

**DOI:** 10.3390/diseases13110356

**Published:** 2025-11-03

**Authors:** Jinguo Wang, Yang Ma, Aixu Duan, Xiaoming Fan

**Affiliations:** 1 College of Nursing, Shanxi Datong University, Datong 037009, China; 03240017@sxdtdx.edu.cn; 2 College of Basic Medicine, Guilin Medical University, Guilin 541004, China; 3 Guangxi Key Laboratory of Tumor Immunology and Microenvironmental Regulation, Guilin Medical University, Guilin 541004, China

**Keywords:** smoking, liver cancer, global epidemiology, disease burden, prevention and control

## Abstract

Liver cancer is a prevalent and highly malignant tumor worldwide, and smoking has been suggested as a potentially significant risk factor, but this association remains understudied and not widely recognized. This study utilized global epidemiological data (1990–2021) from open access databases, analyzing smoking-related liver cancer burden and trends by age, sex, region, and country using mortality, disability-adjusted life years (DALYs), and age-standardized rates (ASRs), with projections for disease burden in 2040. The results show that from 1990 to 2021, the global number of smoking-attributable liver cancer deaths increased (cumulative growth: 67.10%; annual growth rate: 1.63%), while the age-standardized mortality rate (ASMR) declined. Similarly, global DALYs rose (cumulative growth: 49.5%; annual growth rate: 1.32%), yet age-standardized DALY rates (ASDRs) decreased. Significant disparities were observed across gender, age groups, regions, and countries, with higher burdens in males and in regions such as East Asia. Projections indicate that by 2040, both the ASMR and ASDR for smoking-associated liver cancer will decline significantly, particularly among the male population. In conclusion, although the burden of liver cancer related to smoking is on a downward trend, there are still significant demographic and regional differences. Future efforts should prioritize strengthened public health policies, targeted interventions, and further research into the smoking–liver cancer relationship to enhance prevention and control strategies.

## 1. Introduction

Liver cancer remains a major global public health challenge. According to the latest global cancer statistics, there were approximately 865,269 new cases and 757,948 deaths worldwide in 2022 [1]. Accumulating evidence has established smoking as a significant independent risk factor for multiple malignancies. Meta-analyses have demonstrated a dose–response relationship between smoking intensity and the risks of nasopharyngeal carcinoma [2] and gastric cancer [3], while Mendelian randomization studies confirmed that smoking significantly increases esophageal cancer risk [4]. The common mechanisms of cancer include inducing DNA damage, oxidative stress, and immunosuppression, and these processes may also drive the occurrence of liver cancer. 

A systematic analysis based on the Global Burden of Disease Study 2019 showed that smoking and drug abuse, alcohol abuse, and elevated body mass index are the four main risk factors for liver cancer deaths in China [5]. Studies from Poland further indicated that tobacco-related liver cancer contributes to premature deaths [6]. Furthermore, data from a Global Burden of Disease study from 2010 to 2019 indicated that smoking is one of the key risk factors for early liver cancer [7]. In addition, epidemiological evidence and meta-analyses have also reported a positive correlation between smoking and the risk of liver cancer [8]. 

Smoking generates over 4,000 chemical compounds and exerts three major adverse effects on the liver: carcinogenesis, immunomodulation, and direct or indirect toxicity. It upregulates the production of pro-inflammatory cytokines, including tumor necrosis factor-α (TNF-α) and interleukins (IL-1β, IL-6), thereby triggering hepatic fibrosis and necro-inflammatory responses, which ultimately increase the risk of hepatocellular carcinoma [9]. In addition, smoking causes tissue hypoxia, further aggravates DNA damage in liver cells, inhibits the activity of CD4+, CD8+ T lymphocytes, and Natural Killer cells, and weakens anti-tumor immune surveillance [10]. Collectively, these findings underscore smoking as a plausible major risk factor for liver cancer, warranting further research to elucidate its role in prevention and control strategies.

While previous studies have investigated smoking-attributable lung cancer [11] and kidney cancer [12] using Global Burden of Disease (GBD) data, a comprehensive analysis specifically focusing on the global, regional, and national burden of smoking-related liver cancer over the extended period from 1990 to 2021 has been lacking. This study utilized the latest GBD 2021 data to fill this gap by providing a detailed analysis of the epidemiological trends of smoking-induced liver cancer. It reveals significant changes in the disease burden compared with 1990, identifies populations and regions at highest risk, and predicts future trends to 2040, thereby offering crucial evidence for targeted public health interventions.

## 2. Methods

### 2.1. Data Source

This study utilized data retrieved from the Global Health Data Exchange (GHDx, http://ghdx.healthdata.org/, accessed on 15 June 2024) query tool, which hosts the Global Burden of Disease (GBD) 2021 dataset. Our analysis focused on estimating the burden attributable to “smoking” for “liver cancer,” using mortality and disability-adjusted life years (DALYs) as core metrics. Estimates were derived and analyzed across 21 geographical regions, 5 socio-demographic index (SDI) categories, and 204 countries and territories. 

### 2.2. Definition

Smoking: The regular, daily practice of inhaling smoke from burned tobacco, which excludes occasional use and electronic nicotine delivery systems (e-cigarettes).

Liver Cancer: Primary malignancies of the liver, comprising pathological types such as hepatocellular carcinoma, intrahepatic cholangiocarcinoma, and mixed hepatocellular-cholangiocarcinoma.

Deaths: The number of cause-specific fatalities recorded during a defined period.

Disability-Adjusted Life Years (DALYs): A measure of overall disease burden, expressed as the sum of years of life lost due to premature death and years lived with disability.

Age-Standardized Mortality Rate (ASMR): The mortality rate per 100,000 individuals, calculated using a standard population age distribution to enable comparisons across populations with differing age structures.

Age-Standardized DALY Rate (ASDR): The DALY rate per 100,000 persons, similarly standardized for age.

Estimated Annual Percentage Change (EAPC): A metric describing the average annual percentage change in a rate over a specified time interval, used here to analyze trends in ASMR and ASDR. It was calculated by fitting a linear regression model to the natural logarithm of the ASRs: ln(ASR) = α + βx + ε, where x is the calendar year. The EAPC was then computed as 100 × (exp(β) − 1). A positive EAPC with its 95% confidence interval (CI) above zero indicates an upward trend, while a negative EAPC with its 95% CI below zero indicates a downward trend.

GBD Regional Classification: The Global Burden of Disease 2021 study categorizes the world into 21 geographical regions—such as Central Asia, Central sub-Saharan Africa, Central Latin America, Oceania, and Western Europe—and aggregates data from 204 countries and territories.

Age Stratification: The study population is divided into 20 consecutive age groups, starting from under five years old and progressing in five-year intervals up to 95 years and older.

Socio-demographic Index (SDI): A composite indicator of development status that synthesizes average income per capita, educational attainment, and total fertility rate. Ranging from 0 (lowest) to 1 (highest), the SDI value reflects a region’s overall socio-economic development, which is strongly associated with its population health outcomes.

### 2.3. Statistical Analysis

The temporal trend in age-standardized rates (ASRs) for smoking-related liver cancer (1990–2021) was quantified by fitting a linear regression model to derive the EAPC and its associated 95% CI. A positive EAPC with a 95% CI exceeding zero signified an increasing trend, while a negative value indicated a decrease. Standard calculation methods for ASR and EAPC were followed as per the existing literature [13]. We assessed the monotonic relationships between burden metrics, EAPCs, and SDI using Spearman’s rank correlation coefficient [14]. Projections of the disease burden through 2040 were generated using a Bayesian age–period–cohort (BAPC) model. The model was implemented with integrated nested Laplace approximations (INLAs) for robust fitting, and 95% uncertainty intervals (UIs) were derived from 1000 simulation iterations. The R programming environment (v4.2.2) served as the platform for all computations and visualizations, applying a two-sided significance threshold of *p* < 0.05.

## 3. Results

### 3.1. Global Burden of Smoking-Related Liver Cancer in 2021

In 2021, 53,053.68 people (95% UI, 18,267.57–88,111.00) died of smoking-related liver cancer (Table 1), an increase of 67.13% compared with 1990; the ASMR was 0.609 (95% UI, 0.21–1.01)/100,000 person-years. In 2021, the number of DALYs related to smoking-related liver cancer was 1,482,896.29 (95% UI, 504,999.55–2,478,905.62), an increase of 49.51% compared with 1990. The ASDR was 16.90 (95% UI, 5.76–28.26)/100,000 person-years (Table 2).

In 2021, the number of male deaths due to smoking-related liver cancer was 48,166.52 (95% UI, 16,663.66–80,568.20), and that of female deaths was 4887.15 (95% UI, 1464.14–8775.50). The number of deaths among males is 9.86 times that among females. The ASMR was 1.49 (95% UI, 0.52–2.39)/100,000 person-years for males and 0.11 (95% UI, 0.03–0.19)/100,000 person-years for females (Table 1). The DALY of males was 1,370,991.56 (95% UI, 467,666.46–2,286,527.40), that of females was 111,904.73 (95% UI, 33,712.72–2,000,080.73), and that of males was 32.34 (95% UI, 1.05–54.07); for females, it was 2.43 (95% UI, 0.73–4.34) (Table 2).

**Figure 1:**
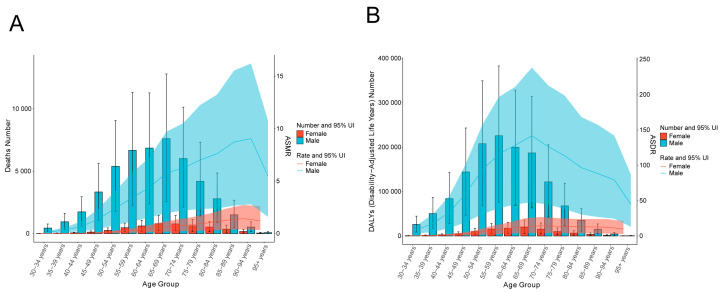
The number and rate of age group deaths and DALYs caused by smoking in liver cancer by gender in 2021. The age-specific number of deaths and mortality rate of liver cancer caused by smoking (**A**). Age-specific quantity and rate of DALYs in liver cancer induced by smoking (**B**). ASMR—age-standardized mortality rate; ASDR—age-standardized disability-adjusted annual rate of life.

In 2021, males and females aged 65 to 69 had the highest number of deaths from liver cancer caused by smoking. Before the age of 65, the number of deaths between males and females increases with age, but after the age of 69, the number of deaths decreases with age. There is a positive correlation between ASMR in females and age. Moreover, the ASMR of liver cancer caused by smoking in both males and females reaches its peak at the age of 90–94 and then gradually decreases (Table 1, Figure 1A). Most DALYs of liver cancer caused by smoking occur in the age group of 55–59 years old. The ASDR of both males and females increased with age until the age of 69, after which it was negatively correlated with age (Table 2, Figure 1B). It is worth noting that in all age groups, the number of deaths and DALYs in males, as well as ASMR and ASDR, were higher than those in females. Moreover, no smoking-induced liver cancer deaths or DALYs were observed in individuals under 30 years old (Table 1 and Table 2, Figure 1).

In addition, liver cancer caused by hepatitis B is the most lethal subtype. In 2021, 24,613 people died (95%UI, 8093–42,004) with DALYs of 773,836 (95%UI, 252,194–1,325,256). Secondly, liver cancer caused by hepatitis C resulted in 12,430 deaths (95%UI, 4105–21,046) and 287,396 DALYs (95%UI, 95,249–482,856) (Table 1 and Table 2).

**Figure 2:**
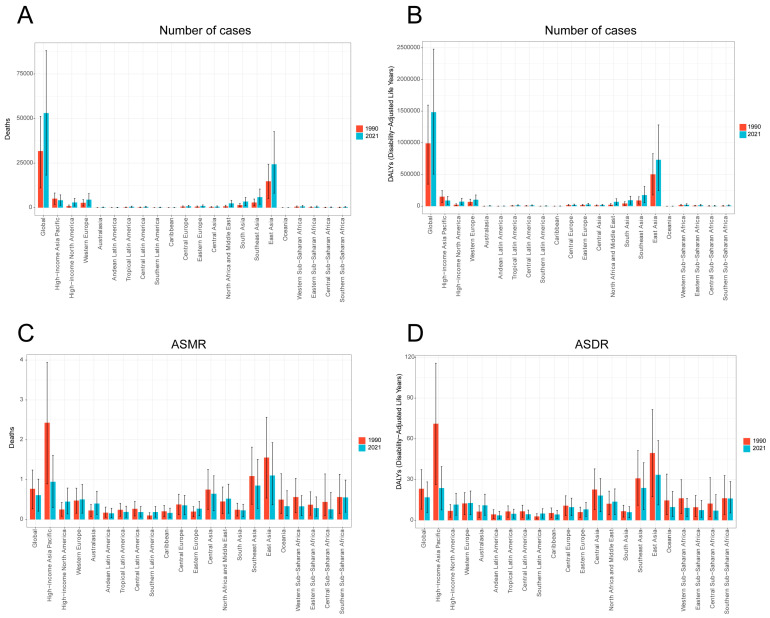
Deaths (**A**), DALYs (**B**), ASMR (**C**), and ASDR (**D**) of liver cancer induced by smoking in 21 regions in 1990 and 2021. ASMR—age-standardized mortality rate; ASDR—age-standardized disability-adjusted annual rate of life.

**Figure 3:**
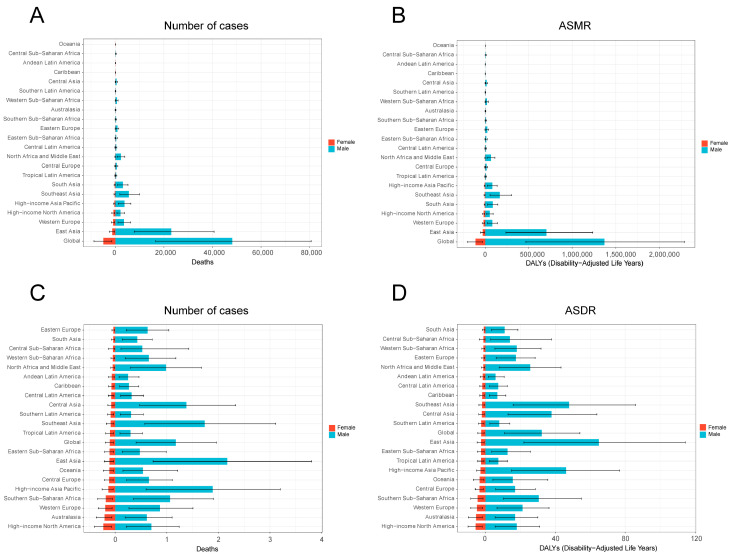
Gender differences in liver cancer induced by smoking in 21 regions in 2021. Gender differences in the number of liver cancer deaths induced by smoking in 21 regions (**A**). Gender differences in DALYs of liver cancer induced by smoking in 21 regions (**B**). Gender differences in ASMR of liver cancer induced by smoking in 21 regions (**C**). Gender differences in ASDR of liver cancer induced by smoking in 21 regions (**D**). ASMR—age-standardized mortality rate; ASDR—age-standardized disability-adjusted annual rate of life.

**Figure 4:**
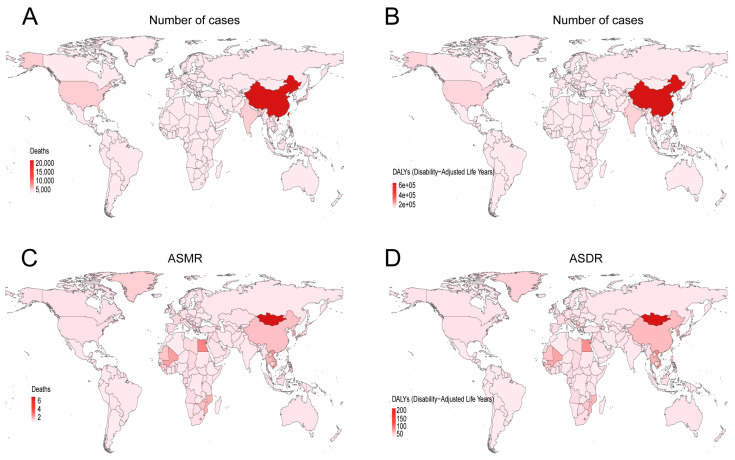
The number of deaths (**A**), DALYs (**B**), ASMR (**C**), and ASDR (**D**) of liver cancer induced by smoking in 204 countries and regions in 2021. ASMR—age-standardized mortality rate; ASDR—age-standardized disability-adjusted annual rate of life.

Among the 21 GBD regions, East Asia exhibited the highest absolute burden of smoking-attributable liver cancer, accounting for the greatest number of deaths and DALYs. There is a significant difference between the high-burden regions in East Asia and the low-burden regions in Western Europe and high-income region of North America, with the former being as much as ten times higher than the latter (Figure 2A,B). The East Asia region also demonstrated the highest age-standardized mortality rate (ASMR) and age-standardized DALY rate (ASDR) (Figure 2C,D). Gender-specific analysis revealed that in 2021, East Asian males had the highest smoking-related liver cancer mortality, DALYs, ASMR, and ASDR among all regions. Notably, across all 21 GBD regions, males consistently showed higher burden metrics than females. In 2021, the number of male deaths was 9.86 times that of females, and male DALYs were 12.25 times higher, underscoring the profound gender-based inequality in the burden of smoking-related liver cancer (Table 1 and Table 2, Figure 3). At the national level, China recorded the largest absolute number of smoking-attributable liver cancer deaths and DALYs in 2021. However, when considering population-standardized rates, Mongolia exhibited the highest ASMR and ASDR globally (Figure 4).

### 3.2. Temporal Trend of Smoking-Related Liver Cancer Burden from 1990 to 2021

Globally, the absolute number of deaths from smoking-related liver cancer increased from 31,744.40 (95% UI, 11,068.23–51,199.91) in 1990 to 53,053.68 in 2021, an increase of 40.17%, but there were some fluctuations. However, ASMR decreased from 0.769 (95%UI, 0.27–1.24) in 1990 to 0.61 (95%UI, 0.21–1.01) in 2021 (Table 1). The absolute number of DALYs increased from 991,836.34 (95%UI, 348,071.52–1,595,195.38) in 1990 to 1,482,896.29 in 2021, representing a growth of 33.1%. Nevertheless, ASDR decreased from 23.19 (95%UI, 8.13–37.31) in 1990 to 16.90 (95%UI, 5.76–28.26) in 2021 (Table 2). The temporal trends in gender subgroups reflected global patterns. Compared with females, the number and rate in males were higher (Figure 1, Table 1 and Table 2).

**Figure 5:**
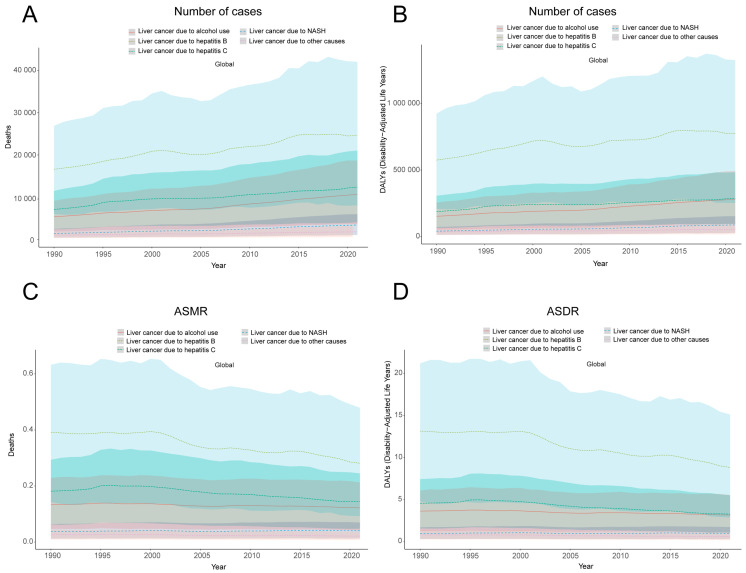
Deaths (**A**), DALYs (**B**), ASMR (**C**), and ASDR (**D**) of various subtypes of liver cancer induced by smoking from 1990 to 2021. ASMR—age-standardized mortality rate; ASDR—age-standardized disability-adjusted annual rate of life.

It is worth noting that from 1990 to 2021, although the number of deaths and DALYs of various subtypes of liver cancer gradually increased, the ASMR and ASDR of liver cancer subtypes showed a gradually decreasing trend. Among them, the reduction in hepatitis C-related liver cancer was the greatest (ASMR EAPC: −1.11, 95% UI, −1.3 to −0.91; ASDR EAPC: −1.34, 95% UI, −1.51–−1.17), followed by hepatitis B-related liver cancer, with a decrease rate similar to that hepatitis C-related liver cancer. Alcohol-related liver cancer decreased slowly (ASMR EAPC: −0.39, 95% UI, −0.45–−0.33; ASDR EAPC: −0.51, 95% UI, −0.57–−0.44), NASH-related liver cancer was the only subtype that increased (ASMR EAPC: 0.23, 95% UI, 0.14−0.31; ASDR EAPC: 0.02, 95% UI, −0.1–0.07) (Figure 5, Table 1 and Table 2).

**Figure 6:**
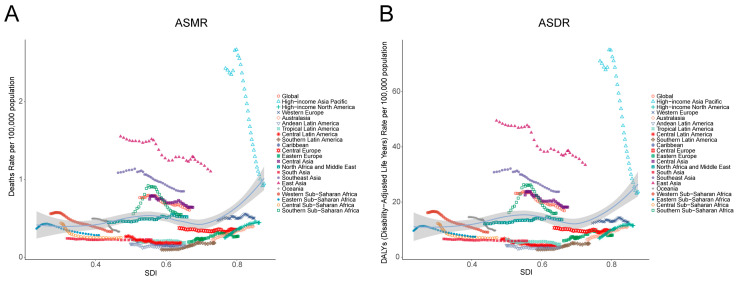
SDI of ASRs in liver cancer induced by smoking in 21 regions from 1990 to 2021. SDI of ASMR in liver cancer induced by smoking in 21 regions from 1990 to 2021 (**A**); SDI of liver cancer ASDR induced by smoking in 21 regions from 1990 to 2021 (**B**); ASMR—age-standardized mortality rate; ASDR—age-standardized disability-adjusted annual rate of life; SDI—social development index.

In addition, from 1990 to 2021, in regions with low SDI levels, changes in SDI had no significant impact on ASMR and ASMR. In regions with medium-level SDI, except for sub-Saharan Africa, the ASMR and ASMR of smoking-induced liver cancer were negatively correlated with the SDI level. In regions with high-level SDI, except for the ASMR of smoking-induced liver cancer in the high-income Asia–Pacific region and the significant negative correlation between ASMR and SDI levels, the changes were not obvious in other regions (Figure 6).

**Figure 7:**
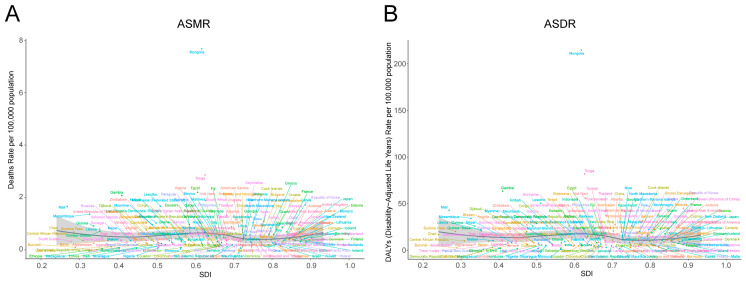
SDI of liver cancer ASRs induced by smoking in 204 countries and regions in 2021. SDI of liver cancer ASRs induced by smoking in 21 regions from 1990 to 2021. SDI of ASMR in liver cancer induced by smoking in 204 countries and regions in 2021 (**A**); SDI of liver cancer ASDR induced by smoking in 204 countries and regions in 2021 (**B**). ASMR—age-standardized mortality rate; ASDR—age-standardized disability-adjusted annual rate of life; SDI—social development index.

At the national level, there was no significant impact on the changes in ASMR and SDI in liver cancer induced by smoking in 2021. In countries with a low SDI level, ASMR and ASDR slightly decreased with the increase in SDI. In countries with a medium SDI level, ASMR and ASDR showed a trend of being high at first and then low with the increase in SDI. In countries with a high level of SDI, ASMR and ASDR increased slightly with the increase in SDI. It is worth noting that Mongolia, which is a country with a medium SDI level, has a significantly higher ASMR and ASDR than other countries (Figure 7).

**Figure 8:**
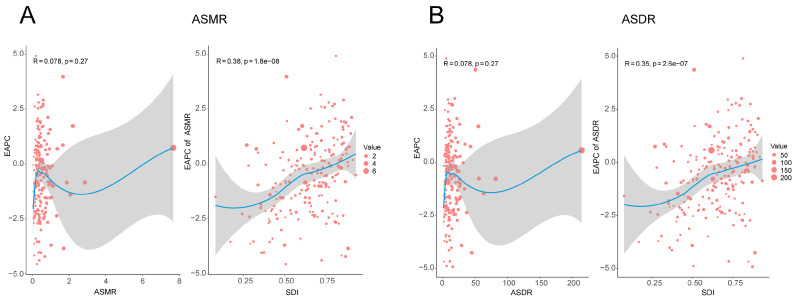
Relationship of ASRs, EAPCs and SDI in liver cancer caused by smoking in 204 countries and regions in 2021. The correlation between EAPC and ASMR as well as SDI in smoking-induced liver cancer in 204 countries and regions in 2021 (**A**); The correlation between EAPC and ASDR and SDI in liver cancer caused by smoking in 204 countries and regions in 2021 (**B**). ASRs: Age Standardization Rate ASMR—age-standardized mortality rate; ASDR—age-standardized disability-adjusted annual rate of life; SDI—social development index.

In addition, from 1990 to 2021, the correlation between EAPC and ASMR and ASDR was not very significant (R = 0.078, *p* = 0.27), while the EAPC of ASMR and ASDR was positively correlated with SDI (ASMR: R = 0.38, *p* = 1.8 × 10^−8^; ASDR: R = 0.35, *p* = 2.6 × 10^−7^). When SDI exceeds 0.5, this relationship becomes more obvious. With the increase in SDI, EAPC tends to be positive more and more (Figure 8).

### 3.3. Future Trends of Global Liver Cancer Burden Related to Smoking from 2022 to 2040

**Figure 9:**
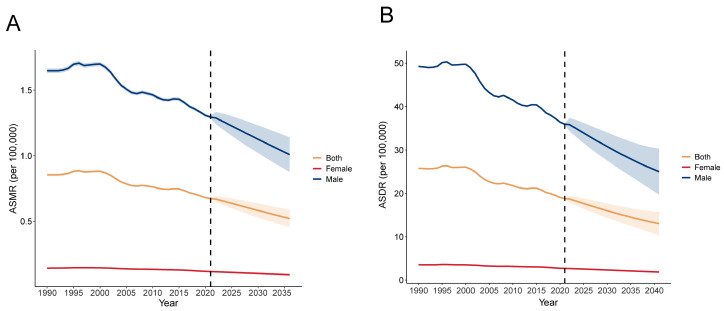
Future global trends of (**A**) ASMR and (**B**) ASDR grouped by gender from 2022 to 2040 based on the APC model. ASMR—age-standardized mortality rate; ASDR—age-standardized disability-adjusted annual rate of life.

According to the estimation of the BAPC model, it is expected that the burden of liver cancer related to smoking will gradually decrease. By 2040, the ASMR and ASDR of smoking-related liver cancer will decline significantly, especially among the male population (Figure 9).

## 4. Discussion

This study utilized the latest GBD 2021 data to comprehensively analyze the epidemiological trends of smoking-related liver cancer worldwide from 1990 to 2021. The results demonstrate that although the ASMR and the ASDR generally showed overall declining trends, the absolute burden of smoking-related liver cancer remains heavy, and there are significant differences in some aspects, such as region, country, gender, age, liver cancer classification, and SDI. Predictions based on the BAPC model show that the burden of diseases will gradually decrease in the future.

From 1990 to 2021, the global number of deaths attributable to smoking-related liver cancer rose by 67.13%, with DALYs increasing by 49.51%. This rise in absolute burden can be largely explained by worldwide population expansion and the demographic shift toward older age structures [15]. Despite these increases, both the ASMR and ASDR exhibited notable declines over the same interval. These favorable trends in standardized rates likely reflected the positive impact of international tobacco control efforts alongside improvements in the diagnosis and management of HCC [16]. A key turning point was the adoption of the World Health Organization Framework Convention on Tobacco Control (WHO FCTC) in 2003, after which numerous countries witnessed a substantial reduction in smoking prevalence [17]. Furthermore, enhanced public health messaging that underscores the dangers of tobacco use has played a complementary role in curbing consumption and mitigating the associated liver cancer burden [18,19].

The disease burden of smoking-related liver cancer shows significant differences in different regions. East Asia bears the heaviest burden. In 2021, the number of deaths in this region accounted for 44.3% of the global total, and DALYs accounting for 44.0%. The number of deaths and DALYs related to smoking-related liver cancer in China is the highest in the world, which may be related to China’s huge population size and the number of smokers [20]. In addition, a positive correlation between smoking and the risk of liver cancer was also confirmed in a large-sample case–control study based on the Chinese population [21]. The ASMR and ASDR in Mongolia are both the highest in the world, which is closely related to its high smoking rate, indoor smoking behavior caused by the cold climate, and limited medical resources [22]. In addition, the rapid economic development of this region has led to changes in lifestyle. Smoking is usually regarded as a symbol of social status, resulting in an increase in the smoking rate [23]. In contrast, in high-income North America and Western Europe, the disease burden was significantly reduced through the early implementation of comprehensive tobacco control measures and a well-established liver cancer screening system [24].

In terms of gender differences, the disease burden of males is significantly higher than that of females. In 2021, the number of male deaths was 9.86 times that of female deaths, and the DALYs were 12.25 times those of female deaths. Furthermore, the ASMR and ASDR of liver cancers induced by smoking in males have consistently been higher than those in females, which might be due to the historically higher smoking rate among males. It is more socially acceptable for males to smoke, while females face more social restrictions on smoking [25]. However, biological differences, including potential hormonal influences on carcinogen metabolism and genetic susceptibility, may also contribute to the disparity and warrant further investigation. Furthermore, in some regions, smoking trends among females are rising. If these trends continue, they could lead to an increased future burden of smoking-related liver cancer in the female population, highlighting the importance of preemptive gender-specific tobacco control measures.

Age distribution analysis shows that in 2021, the number of deaths and DALYs of liver cancer induced by smoking were mainly concentrated in the elderly population. The death toll was the highest in the age group of 70–74-year-olds, and the DALYs were the highest in the age group of 65–69-year-olds. This might be due to the long-term cumulative effects of liver inflammation and DNA damage caused by smoking [26]. In addition, the immune system and repair mechanism of the elderly tend to weaken, usually accompanied by other chronic diseases [26,27]. 

Among the five subtypes of liver cancer related to smoking, liver cancer caused by the hepatitis B virus remains the most serious, followed by liver cancer caused by the hepatitis C virus. From 1990 to 2021, their downward trend was also the most obvious. This overall trend demonstrates the profound effectiveness of specific medical interventions, which operate synergistically with tobacco control. Hepatitis B vaccination and anti-hepatitis C virus treatment confer protection not only against the direct sequelae of viral hepatitis but also indirectly by attenuating the multiplicative risk that smoking poses in individuals infected with the virus. This dual benefit highlights the importance of integrating vaccination and treatment programs with public health efforts aimed at smoking cessation. The slow decline of alcohol-related liver cancer indicates the additive and multiplicative interaction between smoking and drinking [28]. Therefore, alcohol control policies need to be strengthened, especially in areas with a high incidence of alcohol abuse. It is worth noting that NASH-related liver cancer is the only subtype that has risen. This trend is strongly indicative of the escalating global epidemic of metabolic syndrome, characterized by rising rates of obesity, type 2 diabetes, and dyslipidemia, which are key drivers of non-alcoholic fatty liver disease (NAFLD) and its progressive form, non-alcoholic steatohepatitis (NASH) [29]. The increasing burden of NASH-related liver cancer underscores the urgent need to integrate metabolic health promotion and the prevention of obesity into the core of global non-communicable disease control strategies.

The incidence and mortality rates of most cancers show significant socio-demographic differences [30]. In 2021, the Median-Social-Population-Index (SDI) region, especially Mongolia in East Asia, had the highest rates of ASMR and ASDR for liver cancer caused by smoking. This phenomenon may be related to the relatively backward economy and large gap between the rich and the poor in this region. On the one hand, low-income people have more access to tobacco; on the other hand, it is difficult for them to obtain early cancer screening services [31]. In contrast, between 1990 and 2021, the ASMR and ASDR in the high SDI region decreased most significantly, indicating that the high-SDI region has achieved remarkable results in controlling smoking-related cancers. This is mainly attributed to the establishment of well-developed cancer screening programs, improved prevention systems, and advancements in treatment technologies in these countries [32]. However, in light of the current situation that the burden of smoking-related liver cancer persists in areas with medium and low SDI, it is urgent to implement tobacco control projects in these areas. For instance, the MPOWER policy package proposed by the World Health Organization has been proven to effectively reduce tobacco use through measures such as raising tobacco taxes, banning smoking in public places, and large-scale media publicity [33]. In low- and middle-income countries, community interventions that combine health education and convenient smoking cessation services have also shown good results [34]. Our research findings emphasize the necessity of implementing targeted interventions in regions with a high burden of smoking-related liver cancer such as East Asia and Mongolia, for instance, integrating smoking cessation services into the primary health care system and enhancing public awareness through local media to alleviate the future disease burden.

This study has several limitations that should be acknowledged. First, the quality and completeness of the source data vary across countries and regions, particularly in low- and middle-income areas, as in parts of sub-Saharan Africa and South Asia, where the underreporting or misclassification of liver cancer etiology and smoking status may occur. Second, a key challenge in attributing the full burden of liver cancer solely to smoking lies in the frequent co-occurrence of other major risk factors, such as alcohol consumption, chronic hepatitis B and C infections, and metabolic syndromes. The GBD study aims to independently estimate the population-attributable fraction for each risk factor based on theoretical minimum risk exposure levels and relative risks. However, potential synergistic interactions, for instance, between smoking and alcohol use, mean that the independent burden of smoking may be partially conflated. Future Mendelian randomization studies and well-designed prospective cohorts with detailed adjustment for confounders are needed to further clarify the independent causal role of smoking in hepatocarcinogenesis. Additionally, it is noteworthy that the GBD 2023 dataset was released during the final revision of this manuscript. Although our analysis is based on the robust and comprehensive GBD 2021 data, future studies incorporating GBD 2023 estimates would be invaluable to confirm these trends and provide further updates on the global burden of liver cancer attributable to smoking beyond 2021.

## 5. Conclusions

In conclusion, although the age-standardized rate of smoking-related liver cancer worldwide has declined, its absolute burden remains severe. With the intensification of social aging, the increase in age is associated with a sharp rise in the burden of smoking-related liver cancer. Meanwhile, global collaboration should be strengthened to further reduce the burden of diseases.

## Figures and Tables

**Table 1:** The global number, age-standardized rate, and changing pattern of deaths attributable to smoking-related liver cancer in 1990 and 2021.

		1990		2021		1990–2021	
		Number of Death Cases (95% UI)	ASDR/100,000 (95% UI)	Number of Death Cases (95% UI)	ASDR/100,000 (95% UI)	EAPC (95% CI)	Trend
Global		31,744.398 (11,068.228–51,199.905)	0.769 (0.268–1.241)	53,053.676 (18,267.574–88,110.999)	0.609 (0.210–1.012)	−0.84 (−0.96– −0.73)	Down
Gender	Male	29,040.31 (10,240.20–46,715.17)	1.49 (0.52–2.39)	48,166.53 (16,663.66–80,568.20)	1.17 (0.41–1.96)	−0.86 (−0.98– −0.75)	Down
	Female	2704.09 (868.17–4637.61)	0.128 (0.04–0.22)	4887.15 (1464.14–8775.50)	0.11 (0.03–0.19)	−0.63 (–0.75– −0.52)	Down
Age group	30–34	514.76 (172.79–863.89)	0.13 (0.04–2.84)	463.74 (158.64–5855.98 )	0.08 (0.03–1.24)	−2.82 (−3.39– −2.25)	Down
	35–39	1160.10 (399.51–1910.09)	0.33 (0.11–4.07)	977.10 (331.27–9451.67)	0.17 (0.06–2.12)	−2.60 (–2.93– −2.26)	Down
	40–44	2000.17 (710.04–3274.25)	0.70 (0.25–5.11)	1806.27 (598.51–12,007.16)	0.36 (0.12–3.03)	−2.35 (−2.60– −2.10)	Down
	45–49	2607.94 (948.61–4257.31)	1.12 (0.41–6.16)	3462.42 (1128.39–12,343.67)	0.73 (0.24–3.86)	−1.33 (−1.63– −1.03)	Down
	50–54	3782.14 (1412.90–6045.76)	1.78 (0.66−6.61)	5656.14 (1878.91–14,152.70)	1.27 (0.42−5.13)	−0.95 (−1.13– −0.78)	Down
	55–59	4728.26 (1675.97–7546.34)	2.55 (0.90–5.01)	7148.62 (2425.84–47,974.11)	1.81 (0.61–3.34)	−1.09 (−1.20– −0.99)	Down
	60–64	5172.44 (1800.88–8206.44)	3.22 (1.12–4.96)	7475.55 (2601.19–5830.23)	2.34 (0.81–6.66)	−1.08 (−1.25– −0.92)	Down
	65–69	4672.00 (1651.33–7612.81)	3.78 (1.34–4.31)	8417.42 (2841.35–13,334.20)	3.05 (1.03–7.29)	−0.98 (−1.13– −0.70)	Down
	70–74	3351.73 (1146.18–5600.29)	3.96 (1.35–6.34)	6803.65 (2380.09–59,070.12)	3.31 (1.16–3.60)	−0.85 (−1.03– −0.67)	Down
	75–79	2178.10 (713.96–3645.89)	3.54 (1.16–2.54)	4816.61 (1612.02–25,435.19)	3.65 (1.22–5.28)	−0.05 (−0.29– −0.20)	Down
	80–84	1028.69 (330.22–1770.76)	2.91 (0.68–0.93)	3324.78 (1028.86–14,135.43)	3.80 (1.17–6.44)	1.10 (0.85– 1.35)	Up
	85–89	430.49 (135.54–749.92)	2.85 (0.90–0.22)	1869.39 (568.23–20,495.48)	4.09 (0.54–1.24)	1.19 (1.04– 1.34)	Up
	90–94	103.58 (31.51–184.89)	2.42 (0.74–0.54)	700.33 (213.57–794.46)	3.91 (1.19–0.13)	1.41 (1.30– 1.53)	Up
	95+	14.00 (4.19–25.82)	1.38 (0.41–1.14)	131.67 (37.63–1671.88)	2.42 (0.69–0.30)	1.66 (1.53– 1.80)	Up
Liver cancer subtypes	Liver cancer due to NASH	1478.70 (481.08–2539.93)	0.037 (0.01–0.06)	3461.05 (6021.55–1111.96)	0.04 (0.01–0.07)	0.23 (0.14–0.31)	Up
	Liver cancer due to alcohol use	5414.39 (1910.61–9195.91)	0.13 (0.05–0.23)	10,700.49 (3659.14–18,701.10)	0.12 (0.04–0.21)	–0.39 (–0.45–−0.33)	Down
	Liver cancer due to hepatitis B	16,626.05 (6069.01–26,870.83)	0.39 (0.14–0.63)	24,612.92 (8093.26–42,003.77)	0.28 (0.09–0.48 )	−1.05 (−1.17– −0.93)	Down
	Liver cancer due to hepatitis C	7132.23 (2477.06–11,518.32)	0.18 (0.06–0.29)	12,429.99 (4105.23–21,046.42)	0.14 (0.05–0.24)	−1.11 (−1.30–−0.91)	Down
	Liver cancer due to other causes	1093.04 (393.63–1877.90)	0.03 (0.01–0.05)	1849.23 (646.42–3235.51)	0.02 (0.01–0.04)	−0.73 (−0.82–−0.65)	Down

NASH—non-alcoholic steatohepatitis.

**Table 2:** The global number, age-standardized rate, and changing pattern of DALYs.

		1990		2021		1990–2021	
		Number of Death Cases (95% UI)	ASDR/100,000 (95% UI)	Number of Death Cases (95% UI)	ASDR/100,000 (95% UI)	EAPC (95% CI)	Trend
Global		991,836.337 (348,071.515–1,595,195.380)	23.191 (8.129–37.308)	1,482,896.286 (504,999.552–2,478,905.619)	16.903 (5.755–28.259)	−1.121(−1.241–−1.001)	Down
Gender	Male	923,139.30 (326,907.28–1,480,638.48)	44.33 (15.67–71.22)	1,370,991.56 (467666.46–2286527.40)	32.341 (11.05–54.07)	−1.123 (−1.244–−1.003)	Down
	Female	68,697.04 (22,596.93–117,541.21)	3.19 (1.05–5.46)	111,904.73 (33,712.72–200,080.73)	2.43 (0.73–4.34)	−0.86 (−0.96– −0.76)	Down
Age group	30–34	29,794.10 (9996.51–50,050.01)	7.73 (2.59–12.99)	26,889.20 (9195.48–46,104.60)	4.45 (1.52–7.63)	−2.82 (−3.39– −2.25)	Down
	35–39	61,565.66 (21,211.41–101,339.99)	17.48 (6.02–28.77)	51,943.86 (17,618.11–88,977.87)	9.26 (3.14–15.86)	−2.60 (−2.93– −2.26)	Down
	40–44	96,291.25 (34,185.99–157,664.06)	33.61 (11.93–55.03)	86,967.04 (28,806.14–147,039.89)	17.38 (5.76–29.39)	−2.35 (−2.60– −2.11)	Down
	45–49	112,636.42 (40,962.00–183,920.08)	48.51 (17.64–79.21)	14,9559.58 (48,725.60–252,855.80)	31.59 (10.29–53.40)	−1.33 (−1.63– −1.03)	Down
	50–54	145,254.65 (54,230.96–232,028.88)	68.33 (25.51–109.15)	217,615.36 (72,308.57–364,295.63)	48.91 (16.25–81.88)	−0.95 (−1.12– −0.77)	Down
	55–59	159,579.74 (56,525.87–254,671.31)	86.17 (30.52–137.51)	241,731.52 (82,014.18–406,240.77)	61.09 (20.72–102.66)	−1.09 (−1.20– −0.98)	Down
	60–64	150,628.76 (52,439.19–239,231.89)	93.79 (32.65–148.95)	217,757.20 (75,803.12–359,553.51)	68.04 (23.68–112.34)	−1.08 (−1.25– −0.92)	Down
	65–69	114,668.30 (40,527.15–186,926.60)	92.77 (32.79–151.22)	206,699.43 (69,767.39–346,901.88)	74.93 (25.29–125.76)	−0.91 (−1.12– −0.70)	Down
	70–74	67,713.64 (23,158.94–113,274.60)	79.98 (27.35–133.80)	137,603.40 (48,070.90−234,461.89)	66.85 (23.35–113.91)	−0.85 (−1.03– −0.67)	Down
	75–79	35,249.11 (11,539.12–59,018.19)	57.26 (18.75–95.88)	77,885.62 (26,070.00–134,848.71)	59.06 (19.77–102.25)	−0.06 (−0.30– 0.19)	Down
	80–84	13,074.15 (4201.56–22,454.81)	36.96 (11.88–63.47)	42,136.62 (13,025.89–73,918.81)	48.11 (14.87–84.40)	1.09 (0.84– 1.34)	Up
	85–89	4356.63 (1369.72–7580.15)	28.83 (9.06–50.16)	18,898.78 (5742.38–33,844.34)	41.33 (10.56–74.02)	1.19 (1.04– 1.33)	Up
	90–94	908.15 (275.71–1618.18)	21.19 (6.43–37.76)	6139.53 (1873.62–11,164.60)	34.32 (10.47–62.41)	1.41 (1.30– 1.53)	Up
	95+	115.78 (34.57–213.38)	11.37 (3.40–20.96)	1069.15 (305.54–2189.27)	19.62 (5.61–40.17)	1.59 (1.45– 1.72)	Up
Liver cancer subtypes	Liver cancer due to NASH	41,635.59 (13,881.89–71,836.25)	1.00 (0.33–1.71)	87,848.06 (28,384.64–153,226.93)	1.00 (0.32–1.74)	0.02 (–0.1– 0.07)	Up
	Liver cancer due to alcohol use	152,660.03 (53,320.11–254,128.01)	3.64 (1.27–6.08)	280,207.22 (94,450.64–493,221.35)	3.17 (1.07–5.57)	−0.51 (−0.57– −0.44)	Down
	Liver cancer due to hepatitis B	574,319.57 (208,112.37–923,069.70)	13.15 (4.77–21.19)	773,836.16 (252,193.88–1,325,255.78)	8.83 (2.88–15.12)	−1.34 (−1.47– −1.21)	Down
	Liver cancer due to hepatitis C	187,776.50 (65,141.13–306,686.18)	4.57 (1.58–7.44)	287,396.22 (95,249.47–482,855.80)	3.29 (1.09–5.52)	−1.34 (−1.51– −1.17)	Down
	Liver cancer due to other causes	35,444.63 (13,154.74–60,361.64)	0.82 (0.30–1.39)	53,608.62 (18,727.27–93,654.99)	0.61 (0.21–1.06)	−1.02 (−1.11– −0.92)	Down

NASH—non-alcoholic steatohepatitis.

## Data Availability

These data were derived from the following resources available in the public domain: [GBD 2021, http://ghdx.healthdata.org/].
